# Experiences of self-identification, diagnosis and support for adults seeking a recognition of tic disorders in the United Kingdom

**DOI:** 10.1017/S1463423626100905

**Published:** 2026-02-18

**Authors:** Danni Phoenix-Kane, Saskia Keville, E. Bethan Davies, Amanda Ludlow

**Affiliations:** 1 University of Hertfordshire, School of Health, Medicine and Life Sciences, UK; 2 University of Nottingham, School of Medicine, UK

**Keywords:** adulthood, diagnosis, recognition, self-identification, tics, tic disorder

## Abstract

**Aims:**

This study aimed to examine the experiences of self-identification, diagnosis, and support for adults with tic disorders (including Tourette Syndrome) in the United Kingdom (UK).

**Background:**

Traditionally viewed as a neurological disorder of childhood-onset, tic disorders have been observed to remit or persist, often in a milder form, into adulthood. However, the reappearance of problematic symptoms after periods of asymptomatic latency might be more common than previously recognized. The medical exposure and standardization of clinical practice for primary adult-onset or non-typical adult-presenting cases of tic disorders is currently limited and poses barriers to diagnosis.

**Methods:**

An online survey of 42 adults with self-identified tic symptomology explored their tic recognition and journey of attaining a confirmed diagnosis and/or self-identifying after the age of 18 in the UK.

**Findings:**

No significant differences were found between adult and childhood-onset cases. Elevated scores on the Acceptance and Action Tic Specific Questionnaire (AAQ-T) correlated with higher overall frequency, intensity, and severity of motor tics from the Adult Tic Questionnaire (ATQ). The AAQ-T was also shown to negatively correlate with increasing age. Nearly all adults expressed dissatisfaction with the diagnostic process, especially regarding information provided and lack of post-diagnostic support. Those who self-identified quoted fear of dismissal, long waiting lists and lack of understanding from clinicians as reasons for not seeking a formal diagnosis. Overall, the results emphasize the importance for a standardized improved comprehension of tic conditions in healthcare including how to best support adults seeking recognition later in life.

## Introduction

Tic disorders (including Tourette Syndrome (TS) and Chronic Motor and/or Phonic/Vocal Disorder) are typically conceptualized as a childhood contained disorder, where symptoms often diminish over time in adolescence and adulthood to non-clinical or manageable levels (Bloch and Leckman, 2009). However, a growing body of research and accounts of those with lived experiences, challenges the typical view of the clinical course of tics across the lifespan, indicating that tics may endure longer, in a milder more minimal form and that a recurrence of symptoms following asymptomatic latent periods, may be more prevalent than previously recognized (Bruun and Budman, 1997; Schaefer *et al.*, 2017: Black *et al.*, 2021). These periods of latency or temporary amelioration in adulthood may persist for years, contrasting with quicker waxing and waning patterns typically seen in children (Schaefer *et al.*, 2017). Whilst primary adult-onset tics which are not attributed to a recurrence of childhood tics or to another neurologic condition are considered rare, they are recognized to exist (Chouinard and Ford, 2000; Eapen *et al.*, 2002).

The changing nature of tics across the lifespan makes diagnosis particularly difficult, because symptoms can be atypical, fluctuate, or can be masked (Robakis, 2017). For example, the principal symptom of unintended and unvoluntary organic motor (non-rhythmic, rapid movements) and/or vocal tics (vocalizations, often with premonitory urge) which define these disorders are known to wax, wane, fluctuate, and evolve in presentation over time and antecedent contextual/situational exposure (such as emotion, tasks, or stress) and vary between individuals (Conelea and Wood, 2008). Adults may also endure (uniquely or in organic tic combination) with non-organic functional tic-like behaviours (FTLB) which can emulate aspects of organic tics. FTLBs, often given the associated diagnosis of Functional Neurological Disorder (FND), tend to have a later symptom onset, higher female bias, greater tic complexity composition, and more likely to be associated with psychosocial event triggers and familial history of psychiatric disorder (Andersen *et al.*, 2023). Moreover, patients with TS and co-occurring FLTBs are acknowledged as presenting considerable challenges in both diagnosis and treatment posing significant diagnostic and treatment challenges (Cavanna *et al.*, 2025).

The interplay and co-occurrence between health conditions (particularly neurodivergences) also makes it difficult to pinpoint exact onset and potential cause. These co-occurring conditions such as attention deficit hyperactivity disorder (ADHD) and obsessive-compulsive disorder (OCD), are common in individuals with tic disorders, with a large cohort analysis finding only 10% of TS individuals presented without co-occurrences (Cavanna *et al.*, 2011). The co-existence with other conditions may also lead to many females being missed or receiving later tic diagnoses. For example, the co-occurrence of behavioural difficulties in the context of ADHD and autism spectrum disorder (ASD) are more likely to facilitate the detection of tics in males (Garcia-Delgar *et al.*, 2022).

The varying frequency, intensity, and severity of tics also brings further challenges for adults, with compelling evidence indicating diminished quality of life, heightened functional limitations, and greater dissatisfaction with life, compared to the general population (Conelea *et al.*, 2013; Triggiani *et al.*, 2023). Those with tic symptomology in adulthood may also demonstrate the most pronounced clinical manifestations and escalations, which can prove resistant to treatment (Swain *et al.*, 2007). For example, adults with FTLBs also have different prognoses and have been shown to require an altered therapeutic and psychoeducation plan to those with organic tics, being inconsistent or unresponsive to the behavioural and medication usually employed for organic tics (Robakis, 2017; Andersen *et al.*, 2023). As such, getting the right diagnosis is paramount to determining and administering the most effective and favourable treatment and outcome.

Given the potential for exacerbations in clinical presentation over the lifespan, it is critical to understand what diagnosis means and its impact on everyday life. For example, Ross and Rickards (2015) found that while having a TS diagnosis as an adult helped with coping and self-understanding, adults expressed disappointment within the stages of diagnosis, and the lack of clinical knowledge, and post-diagnostic support. There is also the consideration of disclosure – who needs to know, why do they need to know, and the adjustments that may result from disclosure (Love *et al.*, 2023). This is particularly important for conditions considered hidden, given the internal and external stigma individuals may encounter and how this may affect disclosure and support for adults (Fitzgerald, 1998; Valeras, 2010; Kulkarni, 2021).

This current study aimed to understand the journey of self-identification and/or seeking a medical diagnosis of tics in adulthood (aged over 18 years) in the United Kingdom (UK). Specifically, it explored the impact and recognition of their tic presentation, the process and reasoning for obtaining a diagnosis and/or self-identifying with a tic disorder; and for those with a confirmed diagnosis, the post-identification support and validation they received.

## Materials and methods

### Participants and recruitment

Participation was open to adults ≥18 resident in the UK when they sought diagnosis or self-identified with a tic disorder (including Chronic Tic Disorder (CTD), TS and/or FND). The study was conducted in English, and therefore participants had to be able to read and write English to complete the survey.

Four UK-based TS charities (Tourettes Action, Tourette Scotland, Tourette Syndrome – Inclusion in the Community (T.I.C.) Hull, Tourette’s Support N.I.) agreed to feature study advertisements and/or articles about the study on their websites and social media pages. The first author also created accompanying video advertisements which were uploaded to YouTube and disseminated through their social media platforms as alias Tourette Researcher. In all instances, the advertisements were encouraged to be shared to interested parties. All advertisements included a hyperlink to the online survey that was hosted securely by the University of Hertfordshire on Qualtrics. The first page of the online survey consisted of participant study information, which detailed the study purpose, participant criteria, intended participation outcomes, data confidentiality and protection protocol, right to withdraw, ethical approval, and contact details of the researchers and hosting institution. All respondents gave their informed consent to participate online, prior to completing the survey.

The survey was open for two months between May–July 2022. Information was collated anonymously with an average time of ∼20mins for participant completion. Ethics approval for a study cluster, including this study, was obtained from the University of Hertfordshire Health, Science, Engineering and Technology Ethics Committee with Delegated Authority protocol number LMS/PGR/UH/04921.

### Online survey design

The online Qualtrics survey was comprised of both multiple-choice and open-ended questions, with inspiration for the survey’s design taken from Crane *et al.* (2016) and Taylor *et al.* (2022).

The survey was divided into three key sections:

Sociodemographic characteristics: This first section comprised of questions to ascertain the age, gender, UK region residency, ethnic group, and educational attainment of the participants.

Information on the participant’s tics: In the second section, participants were asked to recall their history with tics (such as adult or childhood-onset and estimated age), the nature of their initial symptomatic presentation and any known family history of tic disorders. Participants were also asked to report any co-occurring conditions.

To capture the nature and impact of tics within their current presentation, several tic-specific measurement scales were employed:

- *Adult Tic Questionnaire (ATQ):* A brief self-reported assessment capture of the frequency, intensity, and severity of 27 specific motor and vocal tics over the past week only. Within the questionnaire frequency for each tic was measured with whether they present constant (*C* = 4), hourly (*H* = 3), daily (*D* = 2) or weekly (*W* = 1), and intensity was given a score of between 1 (mild) to 4 (noticeable/severe). Severity was then calculated by a combination of frequency and intensity. The ATQ has proven significant correlation to the gold-standard Yale Global Tic Severity Scale (YGTSS) (Abramovitch *et al.*, 2015).
- *Acceptance and Action Tic Specific Questionnaire (AAQ-T):* An adaptation of the Acceptance and Action Questionnaire as originally described by Hayes *et al.* (2004). This measured experiential avoidance and psychological inflexibility, through rating tics (what do participants think, feel, and do about tics – 0 ‘Not true’ to 4 ‘Very true’) against 15 experiential statements. Total scoring range was 0–60.
- *Daily Interference Scale:* A scale generally conceived from YGTSS (Leckman *et al.*, 1989) that captured the impact participant’s tics have upon their daily life and activity (socially, emotionally, physically, recreationally, and professionally) across 11 domains rated from ‘No interference at all’/‘Not applicable’ (0) to ‘Severe interference’ (4). Total scoring range was 0–44.

The participant’s journey to diagnosis or self-identification: The third section commenced by ascertaining the participant’s diagnostic status of a tic disorder at ≥18 years (whether they received a confirmed diagnosis (privately or through the National Health Service (NHS)), were proceeding or exiting the process before diagnosis, or self-identified with no medical professional confirmation).

This then split the survey and tailored the questions relevant to the respondent’s circumstance:

#### Confirmed diagnosis

For those with a confirmed diagnosis, the estimated time taken to receive a diagnosis and the type of confirmation given (written and/or verbal) was captured. Participants were also asked to confirm the tic disorder diagnostic label given, how they felt when they received the diagnosis, highlight what specialisms they were referred to during the process and detail any post-diagnostic services or information they received. Finally, participants were asked to indicate, using Likert scales, their satisfaction and stress encountered through the diagnostic process.

- 5-point Likert satisfaction scale (‘Extremely dissatisfied’ to ‘Extremely satisfied’) on the manner and understanding of the healthcare professional(s), the information they received about tic disorders, the overall diagnostic journey and post-diagnostic support.
- 4-point Likert stress scale (‘Not at all stressful’ to ‘Very stressful’) on the level of stress they experienced during the diagnostic process.

#### Seeking or exiting the diagnostic process

Participants were asked what tic disorder they were seeking to get diagnosed with, what specialisms they had been referred to when seeking the diagnosis, and what their feelings were on their diagnostic journey. Finally, participants were asked to indicate, using Likert scales, their satisfaction and stress encountered throughout their diagnostic journey.

- 5-point Likert satisfaction scale (‘Extremely dissatisfied’ to ‘Extremely satisfied’) on the manner and understanding of the healthcare professional, the information they received about tic disorders and their overall diagnostic journey.
- 4-point Likert stress scale (‘Not at all stressful’ to ‘Very stressful’) on the level of stress they experienced during the diagnostic journey.

#### Self-identification

Participants were asked what tic disorder they self-identified as having and at what age they made this choice.

All participants were asked to identify any healthcare profession/specialisms they had interacted with to receive a diagnosis or self-identify, what information sources they consulted, and their reasoning and feelings behind seeking, receiving, or exiting diagnosis or self-identifying with a tic disorder.

The final page of the survey consisted of debriefing information, including invitation to follow-up interviews (part of a separate study), and signposting to UK-based tic and wider support organizations and the research team’s contact details.

### Data analysis

The output from the survey was downloaded into Microsoft Excel, and quantitative data analysis was conducted in SPSS V28. Relationships between the AAQ-T and age, daily interference and ATQ total motor/vocal tic frequency, intensity, and severity, as well as age and daily interference were investigated using Pearsons Correlation Coefficient with statistical significance set at *p* ≤ 0.05. Independent *t*-tests between adult-onset and childhood-onset groups were tested for significance (*p* ≤ 0.05) against AAQ-T, daily interference and ATQ total motor/vocal tic frequency, intensity, and severity.

Data screening identified 47 entries needed to be excluded from data analysis due to

either the online survey being incomplete, or they had been given a diagnosis of a tic disorder prior to 18 years. Many of these entries had accessed the online survey but had chosen not to proceed without giving any answers. As such, there was no available demographic data to ascertain whether those adults not taking part could be distinguished from those who had completed the survey.

## Results

### Sociodemographic characteristics

The final sample (Table 1) consisted of 42 participants aged 18–68 (*M* = 36 years; *SD* =13.39). The sample had a gender split of 52.4% female, 31.0% male, 11.9% non-binary, 2.4% gender-fluid and 2.4% trans (female). In terms of ethnicity, the majority (98%) were white British. 76% of respondents had attained education at secondary school or beyond. The sample was diverse in terms of UK residential regions, with most participants located within England (*N* = 34, 81.0%), with representation from Scotland (*N* = 5, 11.9%) and Wales (*N* = 3, 7.1%). There was no representation from Northern Ireland.

**Table 1.** Sociodemographic characteristics of the participants (*N* = 42)

Gender	*N*	(% out of 42)	Age (*M* = 36.12, *SD* = 13.39)	*N*	(% out of 42)
Female	22	52.4	18–25	10	23.8
Male	13	31.0	26–35	13	31.0
Non-binary	5	11.9	36–45	7	16.7
Gender-fluid	1	2.4	46–55	8	19.0
Trans (female)	1	2.4	56–65	3	7.1
			66–68	1	2.4
UK location	*N*	(% out of 42)	Education (highest attained)	*N*	(% out of 42)
England	34	81.0	No formal education	1	2.4
*South West*	9	21.4	Primary/middle	0	0
*South East*	7	16.7	Secondary school	8	19.0
*East Midlands*	4	9.5	College degree	13	31.0
*West Midlands*	3	7.1	Vocational training	3	7.1
*North East*	3	7.1	Bachelor’s degree	3	7.1
*North West*	3	7.1	Master’s degree	3	7.1
*Yorkshire*	3	7.1	Professional degree	2	4.8
*London*	2	4.8	Doctorate degree	2	4.8
Scotland	5	11.9	Prefer not to say	1	2.4
*Grampian*	2	4.8	Other	6	14.3
*Tayside*	2	4.8	*Nursing SEN*	1	2.4
*Lothian*	1	2.4	*Postgraduate Diploma*	1	2.4
Wales	3	7.1	*HNC*	2	4.8
*South West*	2	4.8	*A-levels*	2	4.8
*South East*	1	2.4			

### Participant’s recognized tic history and co-occurring conditions

Challenging the idea that tics in adulthood only represent a recurrence of childhood tics, 13 participants (31%) reported an atypical onset in adulthood, with the remaining 29 participants (69%) recognizing a traditional – but undiagnosed – onset in childhood. Of those who reported an estimated age of onset (*N* = 37), initiation of adult-onset ranged from 18–62 (*M* = 35.5, *SD* = 12.2) with childhood-onset ranging from 4–17 (*M* = 9.6, *SD* = 4.1). Participants tended to report that motor tics were the first manifestation of tic presentation they noticed (*N* = 21, 50%) compared to 6 participants (14.3%) reporting vocal tics. Most participants (57.1%, *N* = 24) could not link tic disorders to any family history. In fact, only 26.2% (*N* = 11) stated a known familial link with a range of differing potential suspected lineage identified such as mother, father, grandmother, grandfather, uncle, siblings and nieces and nephews.

High levels of co-occurring conditions were observed across the sample, with 88.1% reporting at least one or more additional condition(s) (Table 2). The most commonly reported conditions included anxiety (66.7%), ASD (50.0%), ADHD (50.0%), depression (47.6%) and OCD (33.3%).

**Table 2.** Self-reported co-occurring conditions of participants (*N* = 42)

Co-occurring condition	*N*	(% out of 42)
Anxiety	28	66.7
Attention-deficit hyperactivity disorder (ADHD)	21	50.0
Autism spectrum disorder (ASD)	21	50.0
Depression	20	47.6
Obsessive compulsive disorder (OCD)	14	33.3
Sensory processing disorder	12	28.6
Insomnia	9	21.4
Dyslexia	6	14.3
Dyspraxia	3	7.1
**Other**	16	38.1
*Myalgic encephalomyelitis/chronic fatigue syndrome (ME/CFS)*	3	7.1
*Hypermobility*	2	4.8
*Seizures*	2	4.8
*Functional neurological disorder (FND)*	2	4.8
*Chronic pain*	1	2.4
*Auditory processing disorder*	1	2.4
*Pathological demand avoidance (PDA)*	1	2.4
*Delayed sleep phase syndrome (DSP)*	1	2.4
*Post traumatic stress disorder (PTSD)*	1	2.4
*Borderline personality disorder (BPD)*	1	2.4
*Dyscalculia*	1	2.4

### Participant’s current tic presentation and impact

On the ATQ, participants reported on average the presence of 16 tics (*SD* = 6.53). Participants experienced significantly more motor tics (*M* = 9.14, *SD* = 3.27) than vocal tics (*M* = 7.05, *SD* = 3.74) in the past week at the point of taking the survey (*t*(82) = 2.7, *p* = 0.008). Motor tics were also ranked significantly more frequent (*M* = 23.88, *SD* = 11.43; *t*(82) = 2.4, *p* = 0.017), and severe (*M* = 46.12, *SD* = 19.65; *t*(82) = 2.2, *p* = 0.031) than vocal tics though intensity failed to reach a significant difference (*p* = 0.104 (two-tailed)). Table 3 details the presence, frequency, intensity, and severity of reported tics. The three most frequent motor tics reported were head jerk (90.5%), arm/hand movements (90.5%) and facial grimace (85.7%). For vocal tics, the two most present across the sample were sniffing and words (both at 69.0%), though the next present phrases, echolalia and blocking/stuttering were not far behind (all at 64.3%). Syllables were reported to have the most frequency out of the vocal tics, with other vocal tics ranked as most intense and severe.

**Table 3.** Adult tic questionnaire results for self-reported tics experienced by participants over the past week from survey participation

	Present, *N* (% out of 42)	Frequency, *M* (*SD*)	Intensity, *M* (*SD*)	Severity, *M* (*SD*)
Tics	Motor			
Eye blinking	30 (71.4)	2.53 (0.97)	1.83 (0.79)	4.37 (1.35)
Eye rolling/darting	26 (61.9)	2.72 (0.94)	1.88 (0.88)	4.42 (1.60)
Head jerk	38 (90.5)	2.89 (0.98)	2.68 (0.88)	5.50 (1.64)
Facial grimace	36 (85.7)	2.91 (0.98)	2.58 (0.91)	5.42 (1.75)
Mouth/tongue movements	32 (76.2)	2.81 (0.83)	2.07 (0.91)	4.66 (1.68)
Shoulder shrugs	27 (64.3)	2.67 (0.92)	2.46 (1.03)	5.04 (1.65)
Chest/stomach tightening	23 (54.8)	2.50 (1.10)	2.00 (1.04)	4.39 (1.97)
Pelvic tensing movements	15 (35.7)	2.17 (1.03)	1.60 (0.63)	3.33 (1.63)
Leg/feet movements	30 (71.4)	2.66 (0.90)	2.37 (0.89)	4.77 (1.61)
Arm/hand movements	38 (90.5)	3.14 (0.86)	2.92 (0.88)	5.97 (1.37)
Echopraxia	17 (40.5)	2.19 (1.05)	2.35 (0.86)	4.41 (1.62)
Copropraxia	22 (52.4)	2.57 (1.03)	3.00 (0.89)	5.32 (1.67)
Other motor tics	18 (42.9)	3.13 (0.92)	3.16 (1.12)	5.63 (1.89)
Complex motor combinations (multiple tics at once)	32 (78.2)	2.69 (0.97)	3.09 (0.86)	5.36 (1.76)
**Total motor tics ATQ**		**23.88 (11.43)**	**22.24 (9.87)**	**46.12 (19.65)**
Tics	Vocal			
Grunting	22 (52.4)	2.81 (1.08)	2.32 (0.99)	4.78 (1.88)
Sniffing	29 (69.0)	2.96 (0.84)	2.21 (1.05)	5.07 (1.53)
Snorting	13 (31.0)	2.31 (1.03)	1.93 (0.83)	4.07 (1.54)
Coughing	21 (50.0)	2.60 (0.94)	2.18 (1.05)	4.55 (1.65)
Animal noises	21 (50.0)	1.90 (1.09)	2.29 (1.01)	4.19 (1.75)
Syllables	17 (40.5)	3.20 (1.01)	2.29 (0.99)	5.12 (2.00)
Words	29 (69.0)	2.86 (0.97)	2.83 (0.93)	5.59 (1.59)
Phrases	27 (64.3)	2.65 (0.98)	2.96 (0.90)	5.52 (1.42)
Echolalia	27 (64.3)	2.24 (1.05)	2.54 (0.99)	4.69 (1.67)
Coprolalia	25 (59.5)	2.63 (1.06)	3.04 (1.02)	5.56 (1.78)
Blocking/stuttering	27 (64.3)	2.33 (1.04)	2.67 (1.07)	5.00 (1.66)
Other vocal tics	15 (35.7)	2.86 (0.86)	3.19 (0.91)	5.69 (1.14)
Complex vocal combinations (multiple tics at once)	23 (54.8)	2.27 (1.08)	2.91 (1.04)	5.09 (1.59)
**Total vocal tics ATQ**		**17.45 (12.78)**	**18.36 (11.71)**	**35.81 (23.17)**

For total daily interference (range 0–44), scores ranged from 7 to 40, with a mean score of 24.92 (SD = 9.71; Table 4). The three domains reported as being most affected included social situations (how participants felt the tics impacted on their interaction with others, people’s behaviour towards them and feelings of acceptance) (*M* = 3.00, *SD* = 1.01), work and employment (participants ratings on how their tics impacted their ability to do tasks in their job) (*M* = 2.88, *SD* = 1.09) and ‘mood’ (how it affected their state of mind or feeling) (*M* = 2.64, *SD* = 1.12).

**Table 4.** Rating of impact of participants tics upon their daily life (daily interference scale)

Domain	Daily interference of tics, *M* (*SD*)
General activity	2.26 (1.06)
Mood	2.64 (1.12)
Walking ability	1.68 (1.42)
Normal work (daily work outside the home and housework)	2.56 (1.14)
Self-worth	2.55 (1.29)
Relationships with friends, family, and partners	1.88 (1.33)
Social situations	3.00 (1.01)
School or university studies	2.34 (1.08)
Work and employment	2.88 (1.09)
Sleep	2.32 (1.19)
Enjoyment of Life	2.24 (1.27)
*NB – each domain was rated from 0 (‘No interference at all’) to 4 (‘Severe interference’)*	

Total scores on the Acceptance and Action Tic Specific Questionnaire (AAQ-T) ranged from 7 to 55, with a mean total score of 29.36 (*SD* = 11.74). Just over half (54.8%) had a score ≤30. Table 5 details the statements rated. Pearson’s correlations (two-tailed) revealed experiential avoidance to be significantly associated with age (*r* = −0.376, sig. 0.014), daily interference (*r* = 0.696, sig. <0.001), total motor frequency (*r* = 0.539, sig. <0.001), total motor intensity (*r* = 0.488, sig. 0.001) and total motor severity (*r* = 0.559, sig. <0.001). The total vocal tic frequency, intensity, and severity ATQ scores were not significantly correlated with the AAQ-T.

**Table 5.** Rating of acceptance and action questionnaire – tics

Statement	Statement Rating, M (*SD*)
My life won’t be good until my tics are under control	1.38 (1.15)
My negative thoughts and feelings about my tics mess up my life	1.74 (1.29)
The bad things I think about myself because I tic must be true	1.21 (1.26)
I don’t try out new things if I’m afraid I will tic	1.64 (1.21)
I do all I can to make sure I don’t tic in front of other people	2.31 (1.32)
I often catch myself thinking about how I’ve handled situations in which I’ve ticked and what I would do differently next time	2.02 (1.32)
I can’t stand the feeling/sensation I have right before I tic	2.48 (1.21)
I stop doing things that are important to me when I’m having a bad tic day	2.00 (1.47)
When I feel depressed or anxious about my tics, I am unable to take care of my responsibilities	2.07 (1.40)
I wish I could get my anxiety and worries about my tics under control	2.33 (1.41)
I wish I could wave a magic wand to make all my tics go away	2.19 (1.49)
I feel embarrassed by my tics	2.88 (0.92)
I can’t be a good friend when I feel upset about my tics	0.90 (1.08)
When I compare myself to other people who tic, it seems that most of them are handling their tics better than I am	1.64 (1.35)
Whenever I feel the urge to tic, I tic	2.58 (1.23)
*NB – each statement was rated from 0 (‘Not true’) to 4 (‘Very true’)*	

Independent *t*-tests revealed no significant differences between adult-onset and childhood-onset groups across AAQ-T, total daily interference and ATQ total motor/vocal tic frequency, intensity, and severity. Furthermore, there was no significant differences across the same attributes between any of the gender groups (Male, Female, Non-Binary, Trans).

### Diagnostic or self-identifying status and journey

**Figure 1.**
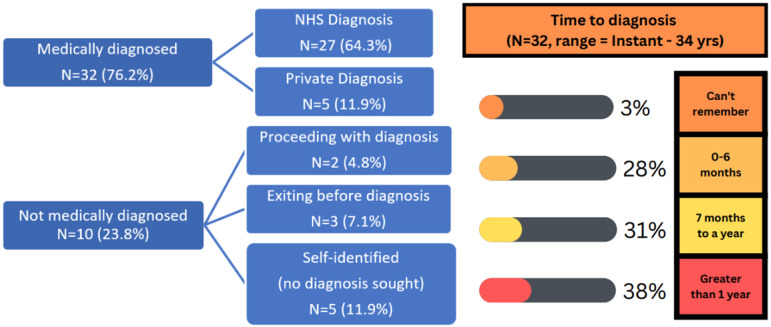
Diagnostic pathway and time to diagnosis.

Of the 42 participants, 76.2% (*N* = 32) were medically diagnosed in adulthood, with the remaining self-identifying 23.8% (*N* = 10) either proceeding with diagnosis (4.8%, *N* = 2), exiting the healthcare interaction before diagnosis (7.1%, *N* = 3) or self-identifying with no diagnosis sought (11.9%, *N* = 5) (Figure 1).

Over half, 52.3% (*N* = 22) of participants interacted with at least two or more healthcare providers including Neurologists (69%), General Practitioners (GP) (50%), Psychiatrists (21%), Clinical Psychologists (10%), Counsellors/Therapists (5%), and Neuropsychiatry/Neuropsychology (7%).

Grouping participants’ responses revealed that participants’ main sources of information they consulted with during self-identification with tics or through seeking a diagnosis was the ‘Internet (incl. NHS website)’ and the ‘[National] Charity (UK)’ (both consulted by *N* = 15 participants, 35.7%) (Table 6).

**Table 6.** Information sources consulted during participants self-recognition (of tic disorder) journey

Source	*N*, % (out of 42)
Internet (incl. NHS website)	15, 35.7
[National] Charity (UK)	15, 35.7
[Local] Charity (UK)	2, 4.8
Academic research	3, 7.1
Education (e.g., University)	2, 4.8
Media influencers (e.g., YouTube, TikTok, Tourette hero)	3, 7.1
Diagnostic criteria (i.e., DSM, ICD)	1, 2.4
Discussion forums (Internet)	2, 4.8
Professionals (e.g., Medical, Therapists)	4, 9.5
Friends/family/personal support network/support groups	3, 7.1
Magazine/newspaper articles	1, 2.4
Didn’t/none/not disclosed	11, 26.2
*NB: Information grouped from written responses. Some responses covered multiple categories.*	

#### Confirmed diagnosis journey (N = 32)

For those with a confirmed diagnosis (*N* = 32), 78.1% (*N* = 25) were diagnosed with TS and 15.6% (*N* = 5) with CTD. One participant was diagnosed with Functional Neurological Disorder (FND) and one participant could not remember the label given (other tic disorder unknown). The diagnosing organization for the confirmed diagnosis cohort was the NHS (84.4% (*N* = 27)) and private (15.6%) (*N* = 5).

The time taken for obtaining a diagnosis was wide ranging, with the majority, 38% of participants having to wait over a year to receive a diagnosis from first interaction, followed by 31% receiving a diagnosis between 7 months to a year afterwards. There was a broad spectrum of reasons for seeking a diagnosis, including interference with their quality of life, to gain a better understanding of themselves and to relieve confusion, reassurance it was not something worse, obtaining validation/evidence for their experiences, protection, and hope of getting medical intervention.

In terms of the type of diagnostics confirmation received, 59.4% of participants received written confirmation (*N* = 19), 21.9% (*N* = 7) received confirmation in both written and verbal form, and 18.8% (*N* = 6) only received verbal confirmation. When asked what participants felt when they received the diagnosis, emotions expressed ranged from positive (‘grateful’, ‘happiness’, ‘relief’, ‘validation/justification’, ‘understanding’) (reported in 75% of participant accounts) to negative (‘loneliness/isolation’, ‘anger at not being understood sooner’, ‘sadness/upset’, ‘disappointed’, ‘hard done by’) (34.3% featured in accounts) or ‘indifferent’/’no surprise’ (18.8% featured in accounts).

Most accounts of those with a diagnosis (*N* = 20) reported that they received no support or were subsequently discharged after diagnosis or failed treatment (62.5%). Table 7 details post-diagnostic support offered which featured the offer of medication (25%), direction to information sheets/webpages (15.6%) and referral to other healthcare specialists (12.5%). For the level of stress, they experienced during the diagnostic process (Table 8), most (78.1%) reported the process being ‘Quite stressful’ or ‘Very stressful’ (*N* = 25).

**Table 7.** Post-diagnostic support received (confirmed diagnosis)

Source	*N*, % (out of 32)
Botox	1, 3.1
Counselling	2, 6.3
CBIT (Comprehensive Behavioural Intervention for Tics)	1, 3.1
Support for other co-occurring condition (e.g., ASD, OCD)	2, 6.3
Further specialist referral offered	4, 12.5
Offered medication options	8, 25.0
Referred to [National] Charity (UK)	3, 9.4
Provided information sheet/webpage	5, 15.6
None/discharged	20, 62.5
*NB: Information grouped from written responses. Some responses covered multiple categories.*	

**Table 8.** Satisfaction and stress experienced by those with confirmed diagnosis or who sought diagnosis (proceeding or exiting)

Confirmed diagnosis	***M***, ***SD***
**Satisfaction (‘Extremely dissatisfied’ (1) to ‘Extremely satisfied’ (5))**	
Manner and understanding of diagnosing professional	3.69, 1.38
Information received at diagnosis	2.84, 1.32
Post-diagnostic support	2.28, 1.20
Overall diagnostic process	3.00, 1.27
**Stress (‘Not at all stressful’ (1) to ‘Very stressful’ (4))**	
Stress	3.06, 0.80
**Proceeding or exiting diagnosis**	***M***, ***SD***
**Satisfaction (‘Extremely dissatisfied’ (1) to ‘Extremely satisfied’ (5))**	
Manner and understanding of healthcare professional	2.2, 1.3
Information received about tic disorders	1.6, 0.89
Overall diagnostic journey	1.6, 0.89
**Stress (‘Not at all stressful’ (1) to ‘Very stressful’ (4))**	
Stress	3.2, 0.84

#### Self-identification journey (N = 10)

Within the self-identification cohort (either proceeding or exiting the diagnostic process or self-identifying with no diagnosis sought) the majority identified with having TS (60%, *N* = 6) with other labels featured such as Chronic Vocal or Motor Tic Disorder (*N* = 1 each) and Tic Disorder Not Otherwise Specified (TDNOS) (*N* = 2).

Two participants were currently seeking a diagnosis and had already been waiting for 5 and 8 months. Of those individuals choosing to exit the diagnostic process, all had already been in the diagnostic process for over a year. Reasons given for originally pursuing a diagnosis focused on getting help, medical intervention, and validation. Conversely, those exiting reported that the process was too difficult, lack of trust in medical professionals, lack of ongoing support, being dismissed or symptoms mistaken for anxiety/depression.

Those who self-identified narrated fear of dismissal, lack of time, waiting lists being too long, and lack of understanding from healthcare professionals as reasons to not seek a diagnosis. One participant refused to seek help due to reporting that their tic disorder was a medicine-induced condition. Negative feelings reported by these participants included ‘embarrassment’, ‘sadness’ and ‘worry being seen as faking the condition’. Positive narratives recorded included ‘relief’, ‘seeing recognition as an initial step towards diagnosis’ or considering self-validation as ‘good enough/content with’.

The satisfaction and stress experienced for those proceeding or exiting the diagnostic process (*N* = 5) is highlighted in Table 8. Compared to confirmed diagnosis participants, there was a lower satisfaction on their journey, information received and manner/understanding of healthcare professionals (Table 8). Stress experienced was also, bar one participant, rated between ‘quite stressful’ and ‘very stressful.’

## Discussion

This is the first study to exclusively capture the post-18 self-identification and diagnostic journey of adults with tic disorders in the UK, exploring several aspects of the diagnostic journey and outcomes (from receiving, proceeding, or exiting a formal diagnosis or self-identifying with no diagnosis sought). To our knowledge, this study expands on previously reported literature looking into diagnosis, motivation regarding diagnostic confirmation and tic symptomology of adults in the UK (Ross and Rickards, 2015; Marino *et al.*, 2023); and is in line with similar studies investigating adult recognition, screening, and clinical implications of other neurodiverse and co-occurring conditions, such as ADHD (Goodman *et al.*, 2016; Anbarasan *et al.*, 2020) and ASD (Woodbury-Smith *et al.*, 2005; Lewis, 2016).

Many of the participants (73.8%) identified as having TS and or a CTD. As such very few fell into FND or Tics Not Otherwise Specified diagnoses; which, considering atypical onset and/or atypical clinical presentation, may be surprising. However, it is important to recognize that FTLBs can be challenging to diagnose, especially when they co-occur with other conditions; here it is co-occurrence of tics with other conditions which was present within the majority of participants in our sample. The difficulty in diagnosis of Functional tics is further compounded by the overlap in symptoms with those seen in TS, making it essential to differentiate between the two (Cavanna *et al.*, 2025).

Moreover, despite primary adult-onset of tic disorders being considered rare, 31% of our cohort reported that their tics started in adulthood. For example, the adult-onset group had a mean of 35.5 years (SD = 12.2, range = 18–62), and indeed, 8 out of 13 from this group experienced atypical tic-onset at ≥30 years of age. Our results, however, are unable to distinguish the classification nature of adult-onset, i.e., idiopathic, secondary, or recurrence/unaware of tics in childhood. Like Chouinard and Ford (2000), we found no significant differences in tic presentation features between childhood-onset and adult-onset groups. For participants who declared an estimated age of onset, those in the childhood-onset group for tics had a mean onset of 9.6 with a range of 4–17 and SD of 4.1 which is corroborated by Marino *et al.* (2023) who found similar (onset 9.72 years, SD = 5.54). In terms of tic presentation, the original manifestation of tics was reported to be predominantly motor, and most participants did not recall a familial history of tics.

There was a surprisingly high female to male ratio in the sample (over half), compared to the 4:1 childhood to adolescence ratio found in favour of males in TS (Lichter and Finnegan, 2015). However, the higher levels of females presenting in our sample may also be reflective of the fact that females with TS are less likely to receive a formal diagnosis and/or wait longer to gain a diagnosis (Dy-Hollins *et al.*, 2025). Health professionals also often struggle to distinguish and categorize symptoms and diagnoses when presented with overlapping symptoms of co-occurring conditions (Bonti *et al.*, 2024). As females have a later symptom onset they may also be more likely to present with tics in adulthood, report more negative impairments to their daily life and are less likely to enter into remission (Lichter and Finnegan, 2015; Garris and Quigg, 2021).

The negative impact of tics for all of these adults was most keenly experienced in social situations, work and employment and mood (from the daily interference scale) and embarrassment (from the acceptance and action tic-specific questionnaire) (AAQ-T). This chimes with previous research of adults with tic disorders, showing that compared to adult controls, they have greater social difficulty (Conelea *et al.*, 2013; Malli *et al.*, 2019), higher unemployment rates (Aldred and Cavanna, 2015), and higher reported struggles with mood, anxiety, and depression symptoms (Evans *et al.*, 2016).

Higher psychological inflexibility and experiential avoidance as measured by the AAQ-T was associated with higher scores on the daily interference, higher motor tic frequency, intensity, and severity. Importantly this became less marked with increasing age. This may infer that psychological distress and experiential avoidance may be more keenly felt the more prominent the adults’ motor tic profile is (frequency, severity, and intensity), though this distress and avoidance may taper off with increasing age. Akin to the findings of Taylor and colleagues (2022), individuals with greater tic severity in the current study were associated with having lower psychological resilience.

Concerning the role of medical professionals/specialists, 52.3% of our overall participants interacted with at least two specialists during diagnosis or self-identification. Those who had a confirmed diagnosis were more likely to have seen a greater number of specialists, with the predominant professionals being Neurologists (69%) and GPs (50%). As such, having to go through more than one specialist may provide credence that due to the complexity and medical awareness of tic disorders when presenting atypically in adulthood, participants are more likely encounter all three phases of Geng *et al.* (2019) three-phase framework (first encounter, initial referral, and subsequent referrals). As such the journey may reflect a diagnostic odyssey spanning years and, in some cases, cause patients to become lost and even disengage prior to formal diagnosis. Moreover, this may have implications on access to formal post-diagnostic support or potentially make the patient’s diagnostic and intervention journey cyclical, which is both personally and financially costly. Hall *et al.* (2024) described case studies of two diagnosed young patients with tics that went through multiple specialists, inconsistent differential and reverting diagnoses, and multiple healthcare interactions. For example, Patient A underwent multiple rejected referrals and numerous appointments across services (health psychology, neurology, CAMHS, paediatrics, and a specialist tic clinic), and Patient B experienced similar rejected referrals and ultimately sought private care. The cost to the NHS was £3512 and £1594 (excluding private care) respectively (Hall *et al.*, 2024). As such medical professionals, may need additional support to reduce pathway resource and time for effective intervention.

Additionally, medical specialists did not always feature highly with the adults for information, with many reporting main sources consulted being the ‘Internet (including the NHS website)’ and the ‘[National] Charity (UK)’. Searching the internet for symptoms and answers is now commonplace, with many turning to search engines (‘Dr Google’) to gain health-related information quickly and effectively, to prepare for medical professional consultation or to gain further clarification from GP advice and information given (Kłak *et al.*, 2017; Van Riel *et al.*, 2017). Cavanna and Hale (2017) explored and scored available patient information leaflets online about TS worldwide, discovering that, although a range of information was available, there was a lack of standardization and a wide variation in the quality, accuracy, and comprehensiveness of information. This self-employed online reliance may also be driven by a lack of full-time equivalent availability and retention of medical professionals (particularly Neurologists and GPs), and supply versus demand issues with increasing number of patients seen in parts of the UK (i.e., England) (Nitkunan *et al.*, 2020; Wise, 2022; British Medical Association, 2023).

Even in the cases where pertinent information from a GP is sought, Marino *et al.* (2023) demonstrated that often no information on causes, management, prognosis, and treatment options was given to patients during their first appointment for tics. Malli and Forrester-Jones (2022)’s investigations into stigma and adults with TS underscored several shortcomings in adults’ healthcare interactions and echoes our results: namely, limited awareness and clinical expertise for TS and delays receiving diagnosis and accessing services. Indeed, the key reasoning for participants who self-identified in our study was fear of dismissal, long waiting times and lack of understanding from healthcare professionals. This was further verified by the low satisfaction scores received by the self-identified cohort across questions pertaining to the manners and understanding given by the healthcare professionals. The self-identified cohort also reported a low satisfaction with the information received and overall diagnostic journey, and an overall high stress scoring for their overall diagnostic experience. Although confirmed diagnosis participants also tended to find the overall diagnostic journey stressful, the mean satisfaction for the diagnostician’s professional manner, information received, and overall diagnostic process was significantly higher than self-identified participants.

Adults who were either seeking and/or had a confirmed diagnosis reported that the diagnosis was important for getting an understanding of oneself, to access or explore medical/medicinal interventions and validation. For confirmed diagnosis participants receiving their diagnosis, 38% of our cohort had to wait more than a year (and in our cohort up to 34 years) which is more extreme than previously reported (Marino *et al.*, 2023). When diagnosis was given, the majority of the adults had personally received a written form of confirmation (81.3%). Open ended questions on experiences when receiving a diagnosis, revealed that 75% of accounts expressed positive terms (e.g., ‘gratefulness’, ‘relief’, ‘happiness’, ‘validation’), highlighting the impactful importance having a confirming label can bring in terms of being understood and gaining answers. This corroborates the themes from Ross and Richards (2015), that a TS diagnosis aids peoples’ ability to manage and understand how to live with their condition.

Our results revealed similarity to other studies highlighting that post-diagnostic support to be limited or even absent (Ross and Rickards.,^2015^; Taylor *et al.*, 2022; Marino *et al.*, 2023). For example, 62.5 % received no support or were subsequently discharged after diagnosis or failed treatment. Those who received support were either offered medication (usually caveated) or signposted to other medical services, charity organizations or therapy (such as counselling). Perhaps it is unsurprising that adults with a confirmed diagnosis were shown to have the lowest satisfaction with post-diagnostic support.

The UK’s National Institute for Health and Care Excellence (NICE) current guidelines offer little advice for adults with tics, other than recommending proceeding with psychological therapy before a referral to neurology, and highlighting issues with poor availability, effectiveness and serious side effects of medication and treatment options (NICE, 2019). However, compared to psychoeducational or psych-supportive therapy in adults, findings into the effectiveness of behavioural therapies such as Comprehensive Behaviour Intervention for Tics (CBIT) indicates that CBIT administered individually (Wilhelm *et al.*, 2012) or in groups (Bekk *et al.*, 2023) may yield better and sustained tic reduction, and thus, should be more widely offered as a potential therapeutic pathway. Notably, both the American Academy of Neurology and European Society for the Study of Tourette Syndrome guidelines suggest the importance of counselling about the natural history of tics, screening for psychiatric co-occurrences, psychosocial education, and the importance of behavioural intervention (Martindale *et al.*, 2023). Considering the risks, side effects and absence of clinical trials demonstrating efficacy, the contemplation of first-and second-line pharmacological treatments is usually only encouraged when behavioural interventions prove unsuccessful or are unavailable (Billnitzer and Jankovic, 2020). Yet only 25% of adults included in the Tourette Association of America survey had participated in CBIT despite CBIT’s beneficial impact on tic disorders (Pringsheim *et al.*, 2019; Andrén *et al.*, 2022). Our study identified an even smaller number of adults diagnosed in adulthood being offered this, with only one of our 32 confirmed diagnosis participants mentioning post-diagnostic support in the form of CBIT, with over 25% of our cohort being offered medication.

Further research would be useful to differentiate whether lack of CBIT treatment was due to unavailability or inaccessibility of behavioural therapy for the participant (either geographically or otherwise), compared to whether behavioural therapy would fail in these adult tic presenting cases, and thus medication was offered. Although there is growing research addressing the use of remote online sessions for tics disorders, with promising early results of diminishing tics and overall satisfaction (Hollis *et al.*, 2021), there has been a limited focus on alternative treatments to CBIT for tic-disorders. Furthermore, our study showed younger adults with more prominent motor tics had higher levels of experiential avoidance and associated distress, an effect which reduced with age. This is potentially indicative that a process of acceptance of these tics may emerge over time reducing experiential avoidance and distress for older participants with more prominent motor tics. Consequently, Acceptance, and Commitment Therapy (ACT) (Hayes *et al.*, 1999; Hayes *et al.*, 2012) may be a viable option for this adult population. Research addressing the use of ACT for tic disorders is in its infancy, yet it has gained increasing popularity by clinicians and mental health professionals in recent years, with the central assumption that much of psychopathology is underpinned by experiential avoidance within a wider process of psychological inflexibility, which can be ameliorated through processes such as acceptance (Hayes *et al.*, 2004).

Overall, our findings illuminate the personal and psychological impact of the self-recognition journey of adults with tic disorders. The study highlights the need for more research, clinical and professional understanding to identify tic morphology, and increased awareness of clinical course, disorder impediment and effective therapeutic options available to the adult population. It would also be useful to conduct further research to understand the recognition and evolution of tics for symptomatic adults who undergo various treatment regimens and track whether these interventions lead to genuine tic reduction compared to less-bothersome tic morphology and reductions in tic awareness.

## Limitations

This study aimed to give insight into the journey of those achieving a formal diagnosis and those who self-identify with a tic disorder in adulthood. However, due to the very small size, our study should be treated as indicative. Caution is also raised regarding the reliance of self-report measures rather than clinical confirmation of diagnosis (Haire *et al.*, 2024), particularly given that health professionals struggle themselves to be able to characterize the nature of adult-onset tics and to distinguish between adult-onset or adult-presenting tic disorders and FTLBs. As such further research could investigate the differences, if any, between personal perception and recognition of adult tics compared to clinical recognition and medical observation of them (Martindale *et al.*, 2023).

Furthermore, there are shortcomings in our methods of recruitment, such that we mainly recruited through charities and support groups. Thus, this self-selective sampling method may not have captured a representative sample and led to overinclusion of those individuals who are actively involved in community groups (Bonevski *et al.*, 2014). For example, males have been less likely to engage with online support groups, often identifying more with a passive stance (Hartley and Schultz, 2015).

The nature of the Adult Tic Questionnaire (ATQ) is also limited, as it simply provides a snapshot of what the morphology of tics had been for participants within the past week (Abramovitch *et al.*, 2015). To elicit the nuanced understanding of the disorder threshold (non-bothersome tics compared to bothersome tics) it may be beneficial to do further longitudinal studies or observations to understand how adult tics are personally identified and how they may manifest, evolve, and are affected by internal and external stigma; particularly given these may influence participant presentation to medical professionals and identification of having a disorder (Malli and Forrester-Jones, 2022).

## Conclusion

Although the current findings have highlighted a dissatisfaction and need for further investigation, alongside guidance on effective post-diagnostic and treatment pathways, there is still a lack of available research on the barriers, effectiveness, and feasibility of implementing treatment pathways for tic disorders. A recent protocol by Martindale *et al.* (2023) provides future insights into how current clinical practice diverges from current guidelines and current gaps in care provision.

It is imperative that future research explores and updates clinical guidelines to ensure more effective resolution (especially for non-typical cases, such as adult-onset or when adult tic symptoms worsen). Indeed, as highlighted by Chouinard and Ford (2000), more research should investigate the differentiated use of identification/labelling of the idiopathic, secondary, or recurrent nature of adult-onset cases on medical presentation, how this is recorded in clinical practice, and the effect this may have on treatments offered for resolution. Finally, our findings would be complemented by more in-depth research or case studies of those who choose to self-identify with tic disorders in adulthood and their motivations towards medical identification or avoidance. Ultimately this may improve awareness, public health intervention strategies and availability of information for a medically marginalized group of adults experiencing adult-onset tics.

## Data Availability

The data forming this study is available upon request. Requests should be directed to the corresponding author, Danni Phoenix-Kane - Email: d.phoenix-kane@herts.ac.uk, Tel: 07846132338.
